# TRPV1 controls innate immunity during *Citrobacter rodentium* enteric infection

**DOI:** 10.1371/journal.ppat.1011576

**Published:** 2023-12-18

**Authors:** Michael Cremin, Emmy Xue Yun Tay, Valerie T. Ramirez, Kaitlin Murray, Rene K. Nichols, Ingrid Brust-Mascher, Colin Reardon

**Affiliations:** Department of Anatomy, Physiology and Cell Biology, UC Davis School of Veterinary Medicine, UC Davis, Davis, California, United States of America; Tufts University, UNITED STATES

## Abstract

Mucosal immunity is critical to host protection from enteric pathogens and must be carefully controlled to prevent immunopathology. Regulation of immune responses can occur through a diverse range of mechanisms including bi-directional communication with neurons. Among which include specialized sensory neurons that detect noxious stimuli due to the expression of transient receptor potential vanilloid receptor 1 (TRPV1) ion channel and have a significant role in the coordination of host-protective responses to enteric bacterial pathogens. Here we have used the mouse-adapted attaching and effacing pathogen *Citrobacter rodentium* to assess the specific role of TRPV1 in coordinating the host response. TRPV1 knockout (TRPV1^-/-^) mice had a significantly higher *C*. *rodentium* burden in the distal colon and fecal pellets compared to wild-type (WT) mice. Increased bacterial burden was correlated with significantly increased colonic crypt hyperplasia and proliferating intestinal epithelial cells in TRPV1^-/-^ mice compared to WT. Despite the increased *C*. *rodentium* burden and histopathology, the recruitment of colonic T cells producing IFNγ, IL-17, or IL-22 was similar between TRPV1^-/-^ and WT mice. In evaluating the innate immune response, we identified that colonic neutrophil recruitment in *C*. *rodentium* infected TRPV1^-/-^ mice was significantly reduced compared to WT mice; however, this was independent of neutrophil development and maturation within the bone marrow compartment. TRPV1^-/-^ mice were found to have significantly decreased expression of the neutrophil-specific chemokine *Cxcl6* and the adhesion molecules *Icam1* in the distal colon compared to WT mice. Corroborating these findings, a significant reduction in ICAM-1 and VCAM-1, but not MAdCAM-1 protein on the surface of colonic blood endothelial cells from *C*. *rodentium* infected TRPV1^-/-^ mice compared to WT was observed. These findings demonstrate the critical role of TRPV1 in regulating the host protective responses to enteric bacterial pathogens, and mucosal immune responses.

## Author summary

Neuroimmune communications are vital in regulating the immune response to invading pathogens. Here we show that during a gastrointestinal infection, pain-sensing neuronal fibers can modulate the immune response to recruit neutrophils via the upregulation of cell adhesion molecules on local blood endothelial cells. This research elucidates a novel impact of the ion channel, TRPV1, on host-pathogen interactions in the gastrointestinal tract as well as a potential methodology for modulating the immune response during enteric infections.

## Introduction

Mucosal host defenses in the intestinal tract are the result of many complex interactions between a diversity of cell types. Host protective immune responses to enteric bacterial pathogens are coordinated by a multitude of factors including the nervous system. The intestinal tract is densely innervated by highly specialized nociceptive sensory neurons, that can detect noxious stimuli, including danger-associated molecular patterns (e.g., ATP), inflammation, and bacterial products. Detection of these substances followed by the activation of neurons is due, in part, to the expression of the polymodal nociceptor transient receptor potential cation channel subfamily V member 1 (TRPV1) [1,2]. These nociceptive sensory neurons are capable of inducing activation of other neurons in a classical reflex arc that involves the coordinated release of peptidic neurotransmitters (neuropeptides) locally in the intestine [3–5]. Neuropeptides, including substance P (SP) and calcitonin gene-related peptide (CGRP), have long been described to exert pro- and anti-inflammatory effects respectively [6–8]. For example, SP is well established to increase blood vessel dilation and permeability, in addition to increasing adhesion molecule expression on endothelial cells [7,9,10]. These physiological changes that allow for increased immune cell recruitment into the local tissue environment are the basis of neurogenic inflammation.

These immunologically potent neuropeptides from sensory neurons have a critical role in the response to infection with bacterial pathogens of the lung, skin, small intestine, and colon. Our previous studies demonstrated that the host response to the mouse-adapted attaching and effacing (A/E) bacterial pathogen *Citrobacter rodentium* was significantly reduced in mice with prior ablation of TRPV1^+^ sensory neurons [11]. This enteric bacterial pathogen possesses virulence genes encoded by the locus of attachment and effacement in a pathogenicity island, a type 3 secretion system, and injects bacterial effector proteins [12,13]. These key aspects have made this model fundamental to our understanding of the host cellular and molecular responses to A/E pathogens *in vivo*. Infection with *C*. *rodentium* triggers host-protective IL-22 production from innate lymphoid cells with the recruitment of monocytes and neutrophils early in the course of infection until adaptive immune responses are generated [14–17]. Adaptive immune responses during *C*. *rodentium* infection are characterized by the recruitment of CD4^+^ T cells that produce IFNγ, IL-17A, and IL-22 and B cells which are required for pathogen clearance [18–20]. Previously, we demonstrated that TRPV1^+^ neurons aided the coordination of this host response [11]; however, the precise role of TRPV1 in the host response during *C*. *rodentium* infection was not assessed. Purported expression of TRPV1 has been reported on numerous cell types in the intestinal tract in addition to neurons ranging from intestinal epithelial cells to T cells [21,22]. With this wide range of cell types that express TRPV1, perhaps it is unsurprising that both pro- and anti-inflammatory have been described in models of intestinal inflammation [23]. It is uncertain if these seemingly contradictory data are simply reflective of the biological complexity or the unique models of intestinal inflammation.

Here we assessed the role of TRPV1 in the development of host immune responses during *C*. *rodentium* infection, using wildtype (WT) and TRPV1 knockout (TRPV1^-/-^) mice. Deficiency in TRPV1 significantly increased the bacterial burden at the peak of infection. The increased *C*. *rodentium* burden in TRPV1^-/-^ mice caused increased colonic inflammation and colonic crypt hyperplasia, which was not due to reduced expression of IFNγ, IL-17A, or IL-22, or the recruitment of T cells into the colon producing these host protective cytokines compared to WT infected mice. Interestingly, infection of TRPV1^-/-^ mice with *C*. *rodentium* resulted in significantly fewer colonic neutrophils 10 days post-infection compared to WT mice. This was correlated with a reduction in expression of the neutrophil chemokine *Cxcl6*. Reduced recruitment was not due to a defect in neutrophil maturation in the bone marrow, but instead driven by downregulation of cell adhesion molecules necessary for rolling adhesion and extravasation. Flow cytometry analysis of colonic blood endothelial cells revealed a significant decrease in ICAM-1 and VCAM-1, but not MAdCAM-1 expression in TRPV1^-/-^ mice compared to WT mice. Collectively these data identify a novel role for TRPV1 in the regulation of neutrophil recruitment to the colon during infection with enteric bacterial pathogens.

## Methods

### Ethics statement

All procedures were approved by the institutional animal care and use committee at University of California Davis, in accordance with the Guide for Care and Use of Laboratory Animals. Mice were euthanized by CO_2_ asphyxiation followed by cervical dislocation according to American Veterinary Medical Association guidelines.

### Animals

C57BL/6 (wildtype, WT) and TRPV1^-/-^ mice were originally purchased from The Jackson Laboratory (Bar Harbor, ME) to establish a breeding colony in our vivarium. Male and female TRPV1^-/-^ mice and C57BL/6 mice were maintained in a specific pathogen-free environment and used for experiments at 6 to 8 weeks old. All animals had *ad libitum* access to food and water.

### *Citrobacter rodentium* and bacterial burden quantification

*Citrobacter rodentium*, strain DBS100, was generously provided by Dr. Andreas Baumler (UC Davis, Davis, CA). Bacteria were grown from frozen stocks on MacConkey agar at 37°C and a single colony was grown in LB broth overnight at 37°C. The bacterial suspension was re-grown at 37°C to reach a final infection suspension containing 10^8^ colony-forming units (CFU). Mice were inoculated by oral gavage with 0.1 mL of the bacterial suspension or sterile LB broth. Mice were euthanized on day 10 or 29 post-infection for tissue analyses. *C*. *rodentium* colonization was quantified on day 10 and 29 by homogenization of either fecal pellet or distal colonic tissue in 1 mL PBS and plating serial dilutions onto MacConkey agar. *C*. *rodentium* colonies were counted after overnight growth at 37°C and results are expressed as CFU/g feces or colonic tissue.

### Histology

Distal colon (1 cm) sections were fixed in 10% buffered formalin, and paraffin embedded for cross-sectioning. Sections (6 μm) were cut and stained with hematoxylin and eosin (H&E). Epithelial cell hyperplasia was evaluated using bright-field microscopy at 20X objective, by measuring the crypt length of 20 well-oriented colonic crypts for each mouse using FIJI (Fiji is just ImageJ, NIH).

### Quantitative PCR

Gene expression was measured by quantitative real-time PCR as previously described [11]. Briefly, tissues were homogenized in TRIzol using a bead beater allowing for isolation of RNA. This RNA was then used to prepare cDNA by reverse transcription (iScript, Bio-Rad Hercules CA) in order to conduct real-time PCR using the indicated primer pairs from PrimerBank [24] **(S1 Table)** with SYBR Green master mix (ThermoFisher, Waltham MA). qPCR was conducted on QuantStudio 6 Flex instrument (ThermoFisher, Waltham MA).

### ELISA

Blood was collected by cardiac puncture with a 1 mL syringe attached to a 26-gauge needle and placed into BD Microtainer (Ref# 365967). After 5 minutes at 20,000 g, the top layer of serum was collected and frozen at -80°C. Untreated 96 flat bottom plates were coated overnight at 4°C with capture antibody for IL-6 from Invitrogen kits (Ref# 88-7044-88). According to manufacturer protocols, plates were read on a BioTek Synergy HTX Multi-mode Plate Reader using Gen5 application by the 450 nm and 570 nm wavelengths. 450 nm wavelength was subtracted from the 570 nm wavelength and the standard curve was determined using a 4-paramter logistic fit.

### Immunohistochemistry and confocal microscopy

Paraffin sections of colonic tissue (6 μm) were used for confocal analysis with antibodies raised against specific proteins of interest according to standard protocols [25]. In brief, after slides were de-paraffinized and rehydrated, antigen retrieval was performed in citrate buffer (10 mM, pH 6.0, 30 min., 95°C). After blocking in 5% BSA (w/v) and normal goat serum, samples were incubated in primary antibody overnight (16 h, 4°C). Slides were washed extensively (3 x 5 mins) in TBS-tween20 and incubated in appropriately labeled secondary antibodies (Invitrogen, Carlsbad CA) for 1 h at room temperature, washed, counterstained with DAPI in TBS-tritonX100 0.1% v/v, washed and mounted in Prolong gold (ThermoFisher, Waltham, MA). Staining using anti-mouse CDH1 (E-cadherin) and anti-mouse βIII Tubulin **(S2 Table)** was revealed using a mouse-on-mouse kit according to manufacturer’s instructions (Vector laboratories, Burlingame, CA). Primary and secondary antibodies used are detailed in **S2 Table.** Slides were imaged on a Leica SP8 STED 3X confocal microscope with a 40X 1.3NA objective or a 63X 1.4 NA objective. All areas larger than the field of view of the objective were acquired using a tiling approach, whereby adjacent images were acquired with a 10% overlap, and processed by Imaris Stitcher (Oxford Instruments United Kingdom).

### T Cell enrichment

Inguinal and mesenteric lymph nodes and spleen were sterilely excised from non-treated C57BL/6 mice and placed on a 100 μm filter and dissociated using the plunger of a syringe followed by several washing steps with stain buffer (1X PBS + 2% FBS). Single cell suspension was treated with ACK lysis solution for 5 minutes at room temperature before being washed. Cells were then incubated with Fc block (anti-CD16/32, 10 μg/ml, Tonbo Biosciences, San Diego, CA) for 15-minute on ice. Antibody cocktail was added to surface stain cells for 30 minutes on ice, followed by washing in stain buffer. The following biotinylated antibodies were added to this cocktail at 1:50 dilution: anti-CD161 (clone# PK136, Ref# 30–5941), anti-CD11c (clone# N418, Ref# 30–0114), anti-Ly6G (Clone# RB6-8C5, Ref# 30–5931), anti-TER119 (Clone# TER-119, Ref# 30–5921), anti-CD11b (Clone# M1/70, Ref# 30–0112), and anti-B220 (Clone# RA3-6B2, Ref# 30–0452) from Tonbo Biosciences. Cells were then washed and resuspended in magnetic streptavidin beads (Cat# 557812, BD Biosciences, Franklin Lakes NJ according to the manufacturer’s protocol. Cells were incubated under mild rotation at 4°C for 30 minutes and then the solution volume was brought up to 1 mL total. Tube was placed in BD IMAG Cell Separation Magnet (Cat# 552311 BD Biosciences, Franklin Lakes NJ) for 8 minutes and the negative fraction was taken and placed into a fresh tube on the IMAG. This was again incubated for 6 minutes and repeated one additional time. All negative fractions were combined, and a fraction of the cells were stained for flow cytometry analysis to confirm T cell purity was above 90%.

### T Cell proliferation assay

96-well round bottom plates were coated with (0, 1, 10 μg/mL) anti-CD3ε (clone# 17A2, Ref# 40–0032, Tonbo Biosciences, San Diego, CA) or PBS for 2 hours at 37°C and removed by aspiration. IMAG negatively selected CD4^+^ T Cells were dyed with CellProliferation Dye eFluor450 (Ref# 65-0842-85, eBioscience, San Diego, CA) according to manufacturer’s protocol then plated in 96-well round bottom plates and cultured in the presence of anti-CD28 (0, 4, 10 μg/mL, Ref# 40–0281, Tonbo Biosciences, San Diego, CA) and/ or capsaicin. Cells were incubated in RPMI media with 10% FBS, 2 mM L-glutamine, 50 μM β-mercaptoethanol, and 1% Penicillin/ Streptomycin statically at 37°C 5% CO_2_ for 72 hours before being harvested for flow cytometry analysis. Proliferation index was determined using the Cell Proliferation Cycle tool in FlowJo (Treestar, Eugene OR).

### Isolation of cells from the colon

Lamina propria lymphocytes were isolated using a lamina propria dissociation kit in combination with a gentleMACS tissue dissociator according to the manufacturer’s instructions (Miltenyi Biotec, Gaithersburg, MD). In brief, colons were removed from the euthanized mouse, opened longitudinally, and cut into 0.5 cm long segments. Epithelial cells were removed by gentle agitation (200 RPM) of tissue fragments in HBSS supplemented with 5 mM EDTA and 5% FBS solution (without Ca^2+^ and Mg^2+^), for 20 minutes at 37°C. Tissue fragments were transferred to a gentleMACS C-tube, with HBSS (with Ca^2+^ and Mg^2+^) and MACS digestion enzymes, and incubated while shaking for 30 minutes at 37°C, followed by dissociation by the gentleMACS device. The resulting single cell suspension was passed through a 100 μm strainer, washed extensively, and subjected to staining.

### Bone marrow cell isolation

Wild-type and TRPV1^-/-^ euthanized mice had their right femur removed. Bone was isolated by removing skin, muscle, and fur and then rinsed with PBS. Ends of the bone were cut and a 26-gauge needle and syringe were used to push PBS through the bone and remove the marrow. Cells were then treated with ACK lysis buffer for 5 minutes at room temperature before undergoing cell surface staining.

### Flow cytometry

Staining of cells was performed using a standard protocol. In brief, cells were counted manually by hemocytometer with trypan blue exclusion and dispensed into flow cytometry tubes, centrifuged, and resuspended in staining buffer containing Fc block (anti-CD16/32, 10 μg/ml, Tonbo Biosciences, San Diego, CA) for 25-minute on ice. Antibody cocktail **(S3 Table)** was added to surface stain cells for 30 minutes on ice, followed by washing in stain buffer. Viability was determined using live/dead aqua according to manufacturer’s instructions (ThermoFisher, Waltham MA). All flow cytometry data was acquired on a LSRII (BD Biosciences, Franklin Lakes NJ) using DIVA software, with analysis using FlowJo (Treestar, Eugene OR)

### Intracellular staining

Prior to surface staining, cells were incubated in RPMI media with 10% FBS, 1% Penicillin/ Streptomycin, 2 mM L-glutamine, and BD GolgiPlug (1:500, Cat# 555029, BD Biosciences, Franklin Lakes NJ) for 4 hours at 37°C. To stimulate cells, 1X Cell Stimulation Cocktail (phorbol 12-myristate 13-acetate) was added (eBioscience, San Diego CA). Following surface staining cells were fixed and permeabilized using a BD Cytofix/ Cytoperm Fixation/ Permeabilization Solution kit (Cat# 554714 BD Biosciences, Franklin Lakes NJ) followed by intracellular staining with anti-IFNγ, anti-IL-17A, and anti-IL-22 **(S3 Table)** in 1X BD Perm/ Wash Buffer (Cat# 554723 BD Biosciences, Franklin Lakes NJ).

### TRPV1 mRNA expression determination

Naïve control (LB gavage) and *C*. *rodentium* infected WT and TRPV1^-/-^ mice were euthanized and colons removed. Colons were reduced to single cell suspension as described above and stained with anti-Ly6G, anti-CD3, anti-gp38, anti-CD31, anti-CD45, and Live/ Dead Fixable Aqua followed by sorting (Astrios Cell Sorter, Beckham Coulter, Brea CA). Live single neutrophils (CD45^+^ Ly6G^+^), T cells (CD45^+^ CD3^+^), blood endothelial cells (BEC: CD45^-^ CD31^+^ gp38^-^), lymphatic endothelial cells (LEC; CD45^-^ CD31^+^ gp38^+^), and stromal cells (CD45^-^ CD31^-^) were sorted and processed for qPCR analysis using the Takara CellAmp Direct TB Green RT-qPCR Kit (Cat# 3735A). In brief, cells were washed with CellWash Buffer, and lysed and frozen at -20°C before being thawed and cDNA generated. qPCR was performed using primers specific for the deleted region in TRPV1^-/-^ mice. CT values were first normalized to β-actin and then normalized to vagal ganglion samples as tissue with TRPV1 expression.

### Ussing chamber

Mouse colon was excised, cut along the mesenteric border, and then mounted onto cassettes (Physiologic Instruments) allowing for 0.1 cm^2^ of the mounted colonic tissue to be exposed to 4 mL of circulating oxygenated Ringer’s buffer (115 mM NaCl, 1.25 mM CaCl_2_, 1.2 mM MgCl_2_, 2.0 mM KH_2_PO_4_, and 25 mM NaHCO_3_) at 37°C. To keep the colonic tissues healthy and viable throughout the experiment, 10 mM glucose (Sigma Aldrich, St. Louis, MO) was added to both the serosal and mucosal compartments. Two pairs of electrodes attached to agar-salt bridges were used to monitor the potential difference between both compartments and to inject current during voltage clamping. Recordings and current injection were performed using an automated voltage clamp and Acquire and Analyze software (Physiologic Instruments, San Diego CA). After 20 minutes of equilibration, 88 mg/mL of FITC-labeled dextran (Sigma Aldrich, St. Louis, MO) was added to the mucosal chamber to measure tight junction permeability over time. This was achieved by collecting samples from the serosal compartments every 30 minutes for 2 hours and measuring the respective FITC concentrations on a plate reader (Synergy H1; BioTek, Winooski, VT). Samples with visible FITC leakage in the serosal compartment were omitted. Baseline active ion transport was measured by short-circuit current (Isc) and tight junction permeability was measured by conductance (G).

### Epithelial organoid cell culture & proliferation

Colonic crypts were isolated and cultured as described previously with minor modifications [26]. Mouse colon was dissected out, flushed thoroughly with ice cold PBS, opened longitudinally and then cut into 5 mm-long fragments. Tissue fragments were washed repeatedly in ice-cold PBS and then incubated in chelation buffer (5.6 mM Na_2_HPO_4_, 8 mM KH_2_PO_4_, 96 mM NaCl, 16 mM KCl, 44 mM sucrose, 54.8 mM D-sorbitol, 5 mM EDTA and 0.5 mM DTT) for 30 minutes on ice. Colonic crypts were gradually released from the tissue fragments during 12 rounds of washing in 2% fetal bovine serum and filtering through 70 μm filter mesh, generating 12 fractions. Only fractions 8 to 12 containing enriched colonic crypts were used for downstream experiments. About 200 colonic crypts were seeded in 25 μL Cultrex BME Type 2 (R&D systems, Minneapolis, MN) domes and cultured in Advanced DMEM/ F-12 supplemented with N2, B27, 50 ng/mL EGF, 100 ng/mL Noggin, 500 ng/mL R-spondin, 100 ng/mL Wnt-3an, 1 mM N-acetyl-L-cysteine and 1 μM ROCK inhibitor (Y27632, Millipore, Burlington, MA). Organoid proliferation was assessed with EdU incorporation assay at day 5 post crypt seeding. EdU (10 μΜ) was added to the culture media for 24 h and visualization was performed according to the manufacturer’s instruction with Click-iT EdU Cell Proliferation Kit Alexa Fluor 647 (ThermoFisher, Waltham, MA). Organoids were imaged on a Leica SP8 STED 3X confocal microscope with a 40X objective. The percentage of EdU positive cells was quantified manually with Imaris software.

### Statistics

Statistical analysis of all data was performed using Prism 9.0 (GraphPad, La Jolla, CA) with a Student’s t test, one-way, or two-way ANOVA followed by a post-hoc analysis with Tukey’s multiple comparison test. Individual data points are presented as mean ± standard error of the mean.

## Results

### TRPV1 deficiency increases bacterial burden and colonic pathology during *C*. *rodentium* infection

**Fig 1:**
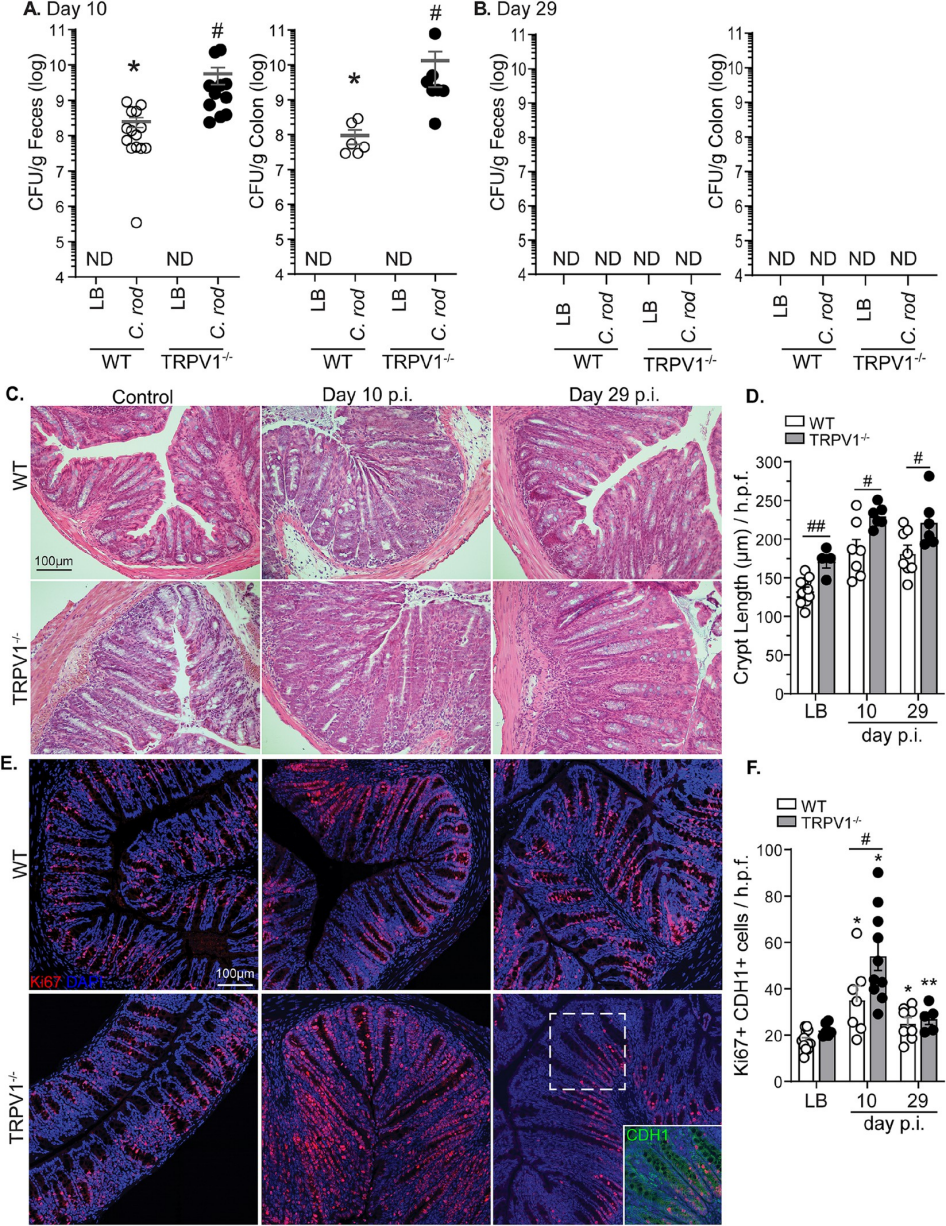
TRPV1^-/-^ mice have an increased bacterial burden and colonic crypt hyperplasia during *C*. *rodentium* infection. Wild-type (WT; open circles) and TRPV1^-/-^ mice (black circles) were infected with *C*. *rodentium* or given vehicle control (LB) by oral gavage. The number of fecal and colonic tissue adherent bacteria were assessed in WT and TRPV1^-/-^ mice at **(A)** 10 days p.i. and **(B)** 29 days p.i. **(C)** Hematoxylin and eosin (H&E) stained paraffin-embedded cross-sections of colon tissue from uninfected control, or 10, 29 days p.i. (Scale bar: 100 μm), with **(D)** crypt length measured using FIJI. **(E)** Confocal images of distal colon tissue from uninfected, 10, or 29 days p.i. mice stained with Ki67 (red), CDH1 (green), and DAPI (blue), with **(F)** quantification of Ki67^+^ CDH1^+^ DAPI^+^ cells. Data are from 7–12 mice per group in 4 separate experiments, and presented as mean ± standard error of the mean: *, *P* < 0.05, **, *P* < 0.01, and ***, *P* < 0.001 vs uninfected mice of the same genotype; #, *P* < 0.05, # #, P< 0.01 compared with WT mice at the same time point; Student’s t test **(A & B)** and two-way ANOVA **(D & F)** with post-hoc analysis using Tukey’s multiple comparisons test. LB, Luria-Bertai; p.i., post-infection.

To determine the role of TRPV1 in the host response to an A/E pathogen *in vivo*, TRPV1^-/-^ and WT mice were administered LB (control) or *C*. *rodentium* by orogastric gavage. Fecal and colonic *C*. *rodentium* were significantly increased in TRPV1^-/-^ compared to WT mice 10 days post-infection (p.i.) **(Fig 1A)**, with both TRPV1^-/-^ and WT mice clearing the infection by 29 days p.i. **(Fig 1B)**. This significant increase in bacterial burden 10 days p.i. in TRPV1^-/-^ mice was associated with increased severity of *C*. *rodentium* induced colonic pathology. As expected, colonic crypt hyperplasia was observed in mice infected with *C*. *rodentium* compared to uninfected controls. This increase was significantly exacerbated in TRPV1^-/-^ compared to WT mice at 10- and 29-days p.i. **(Fig 1C and 1D)**. Infection-induced crypt hyperplasia was due to increased intestinal epithelial cell proliferation as revealed by significantly increased Ki67^+^ CDH1^+^ DAPI^+^ cells 10- and 29-days p.i. **(Fig 1E and 1F)**. Small but significantly increased crypt lengths were observed in uninfected TRPV1^-/-^ compared to WT mice, although there was no increased basal intestinal epithelial cell (IEC) proliferation in uninfected mice.

Although expression of TRPV1 has been reported on many cell types including sensory neurons, intestinal epithelial cells, and immune cells [27–31], non-specific TRPV1 immunoreactivity was observed in TRPV1^-/-^ colons, and was localized to colonic epithelial cells. Neuronal TRPV1-specific immunoreactivity (βIII-tubulin^+^) was only found in WT and not TRPV1^-/-^ colon **(S1A Fig)**. With potential TRPV1 expression on intestinal epithelial cells, we investigated the impact of TRPV1 on epithelial cell proliferation and function. Assessment of colonic permeability by FITC-dextran in Ussing chambers found no significant increase in permeability of TRPV1^-/-^ vs WT mice **(S1B Fig)**. Intestinal epithelial cell proliferation was not to be dependent on TRPV1, as incorporation of the thymidine analogue EdU and organoid diameter was equivalent in WT and TRPV1^-/-^ organoids treated with vehicle or increasing concentrations of the TRPV1 agonist, capsaicin **(S1C Fig)**.

### CD4+ T cell response to *C*. *rodentium* is intact in TRPV1^-/-^ mice

It is well established that the differentiation and recruitment of specific CD4^+^ T cell subsets are required for the control of *C*. *rodentium* infection [32,33]. As TRPV1 has been suggested to enhance proliferation and differentiation of T cells in a cell intrinsic manner [21], we assessed if T cell responses during *C*. *rodentium* infection were altered in TRPV1^-/-^ compared to WT mice.

**Fig 2:**
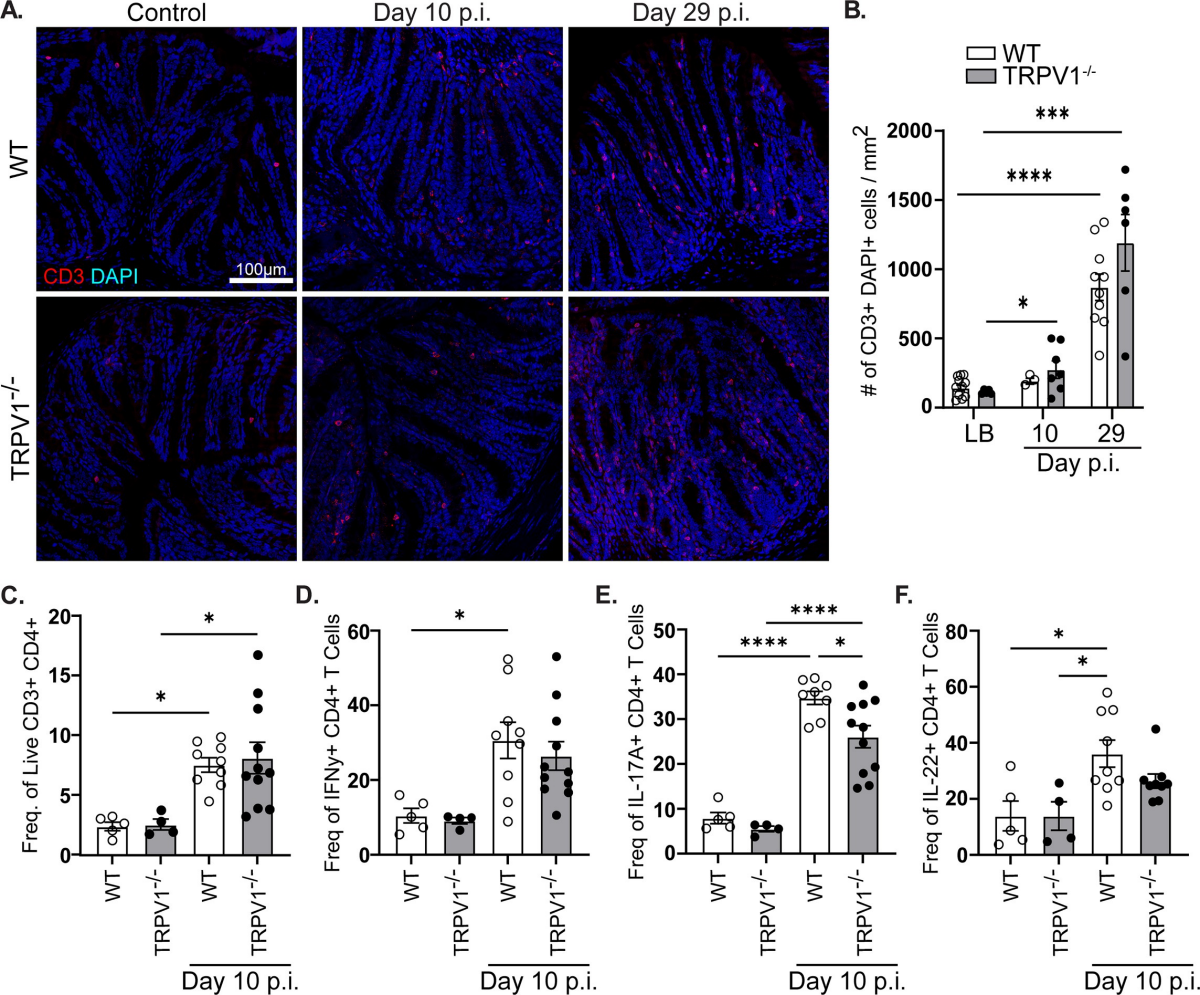
T cell recruitment and cytokine production are unaffected by TRPV1 deficiency during *C*. *rodentium* infection. **(A & B)** Colonic tissue sections were assessed for CD3^+^ (red) DAPI^+^ (blue) T cell infiltration in vehicle control (LB) and *C*. *rodentium* infected wild-type (WT) and TRPV1^-/-^ mice after 10- or 29- days p.i. **(C)** Lamina propria lymphocytes were assessed by flow cytometry to enumerate live CD45^+^ CD3^+^ CD4^+^ T cells and determine the frequency of **(D)** IFNγ^+^, **(E)** IL-17A^+^, or **F)** IL-22^+^ T cells in control and infected WT and TRPV1^-/-^ mice 10 days p.i. Data are presented as mean ± standard error of the mean: *, P < 0.05, **, P < 0.01 and ***, P < 0.001; one-way ANOVA with post-hoc analysis using Tukey’s multiple comparisons test, with 4–11 animals per group. LB, Luria-Bertai; p.i., post-infection.

Enumeration of colonic T cells revealed no significant differences in TRPV1^-/-^ vs WT mice by confocal microscopy after 10- and 29- days p.i. **(Fig 2A and 2B)**. These data were confirmed by flow cytometry analyzing the number of CD3^+^ CD4^+^ T cells present in the lamina propria at baseline and 10-days p.i. **(Fig 2C)**. Intracellular cytokine staining conducted on these lamina propria lymphocytes further revealed no significant difference in the frequency of CD3^+^ CD4^+^ T cells producing IFNγ and IL-22, with a slight but significant decrease in the frequency of IL-17A+ T cells in TRPV1^-/-^ compared to WT mice 10 days p.i. **(Fig 2D–2F)**. However, quantification by qPCR revealed no significant difference in *IFNγ*, *IL-17a*, or *IL-22* expression in the colon of WT or TRPV1^-/-^ mice 10- or 29- days p.i. **(S2A–S2C Fig)**. To investigate if TRPV1 could act in a cell intrinsic manner to regulate T cell proliferation, we assessed the proliferative response of WT and TRPV1^-/-^ CD4^+^ T cells. Stimulation with increasing concentrations of anti-CD3ε, anti-CD28, with and without the TRPV1 agonist capsaicin showed no difference in proliferation **(S2D and S2E Fig)**. Together, these data demonstrate that T cell responses in TRPV1^-/-^ mice remain intact during *C*. *rodentium* infection.

### Lack of TRPV1 causes dysregulation of innate immune responses during *C*. *rodentium* infection

**Fig 3:**
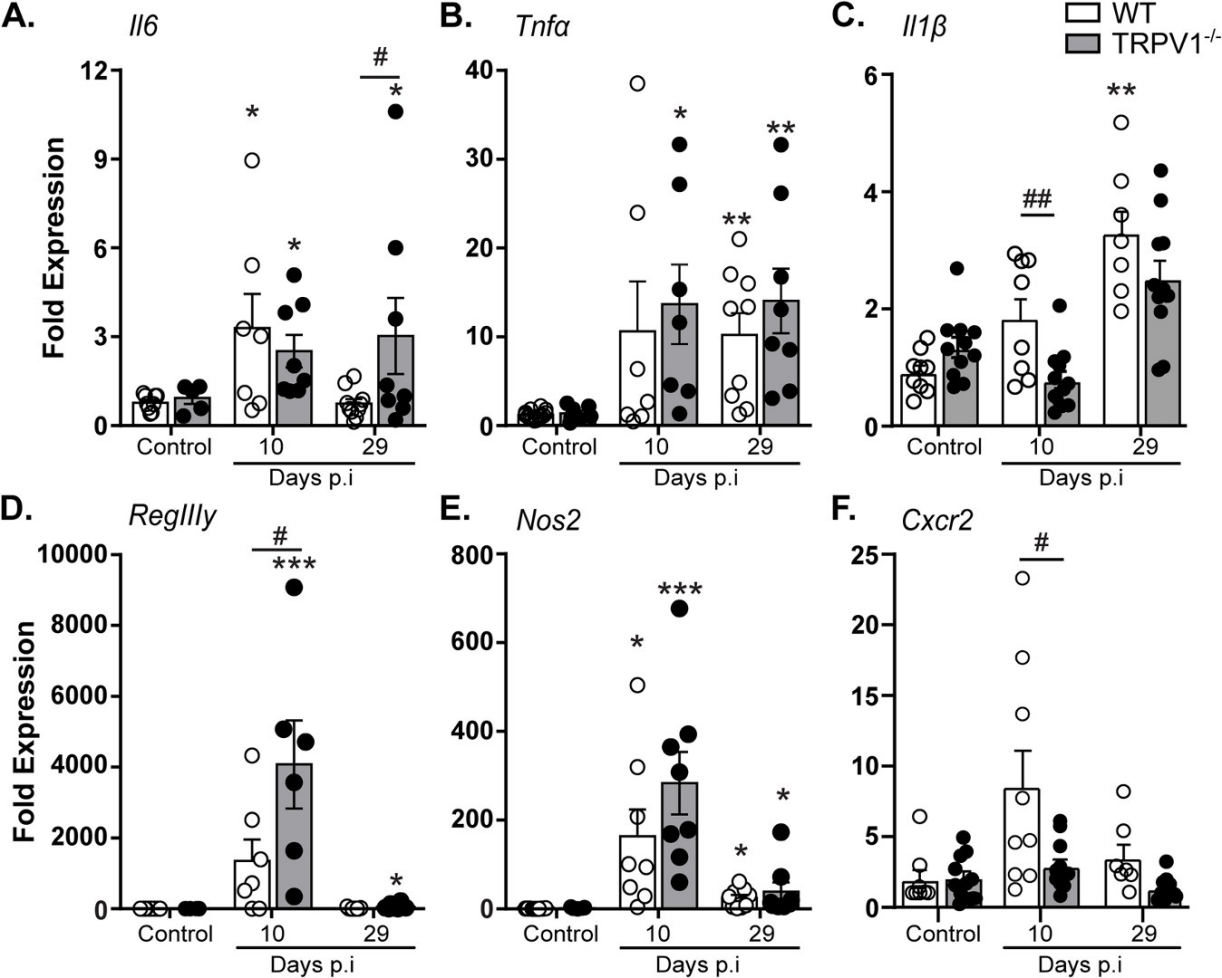
Select innate immune responses are reduced in *C*. *rodentium*-infected TRPV1^-/-^ mice. The host immune response during infection was assessed through qPCR conducted on colonic tissue from control or *C*. *rodentium* infected wild-type (WT) or TRPV1^-/-^ mice. These include the expression of proinflammatory cytokines **(A)** *Il6*, **(B)** *Tnfα*, **(C)** *Il1β*, **(D)** the antimicrobial peptide *RegIIIy*, **(E)** inducible nitric oxide synthase (*Nos2*), and **(F)** the neutrophil chemokine receptor *Cxcr2*. Data are presented as mean ± standard error of the mean, n = 7–13 animals/ group: *, P < 0.05, **, P < 0.01, and ***, P < 0.0001 versus uninfected mice of the respective genotype; #, P < 0.05 and ###, P < 0.0001 compared with WT *C*. *rodentium*-infected mice at the same time point; one-way ANOVA with post-hoc analysis using Tukey’s multiple comparisons test. p.i., post-infection.

With the increased bacterial burden and histopathology in *C*. *rodentium* infected TRPV1^-/-^ mice compared to WT mice without reduced T cell recruitment, we sought to characterize the host innate immune response. Infection of WT and TRPV1^-/-^ mice significantly increased colonic *Il6* and *Tnfα* expression compared to uninfected controls **(Fig 3A and 3B)**. While *Il6* mRNA levels returned to baseline by day 29 p.i. in WT, significantly increased *Il6* was still observed in TRPV1^-/-^ mice at this time **(Fig 3A)**. Expression of *Il1β* was significantly impaired in TRPV1^-/-^ mice at 10 days p.i. compared to WT; however, this difference is diminished by 29 days p.i. **(Fig 3C)**. At day 10 p.i., serum IL-6 was elevated in both WT and TRPV1^-/-^ mice compared to uninfected controls; however, at day 29 there was no detectable serum IL-6 **(S4 Fig)**.

Reflective of the increased *C*. *rodentium* burden, expression of the antimicrobial gene RegIIIγ was significantly increased during infection, and further enhanced in infected TRPV1^-/-^ compared to WT mice 10 days p.i. **(Fig 3D)**. However, altered expression of innate and host protective genes in *C*. *rodentium* infected TRPV1^-/-^ mice was not universal. Although inducible nitric oxide synthase (iNOS, NOS2) expression was induced by *C*. *rodentium* infection, no difference was detected 10- or 29-days p.i. in WT and TRPV1^-/-^ mice **(Fig 3E)**. Interestingly, we found a significant decrease in *Cxcr2* expression, a neutrophil chemokine receptor critical for chemotaxis into the lumen of the gut, in TRPV1^-/-^ mice compared to WT at 10 days p.i. **(Fig 3F)**. These data suggest innate immune response may be altered in TRPV1^-/-^ mice during *C*. *rodentium* infection.

### Colonic neutrophil recruitment during *C*. *rodentium* infection is reduced in TRPV1^-/-^ mice

**Fig 4:**
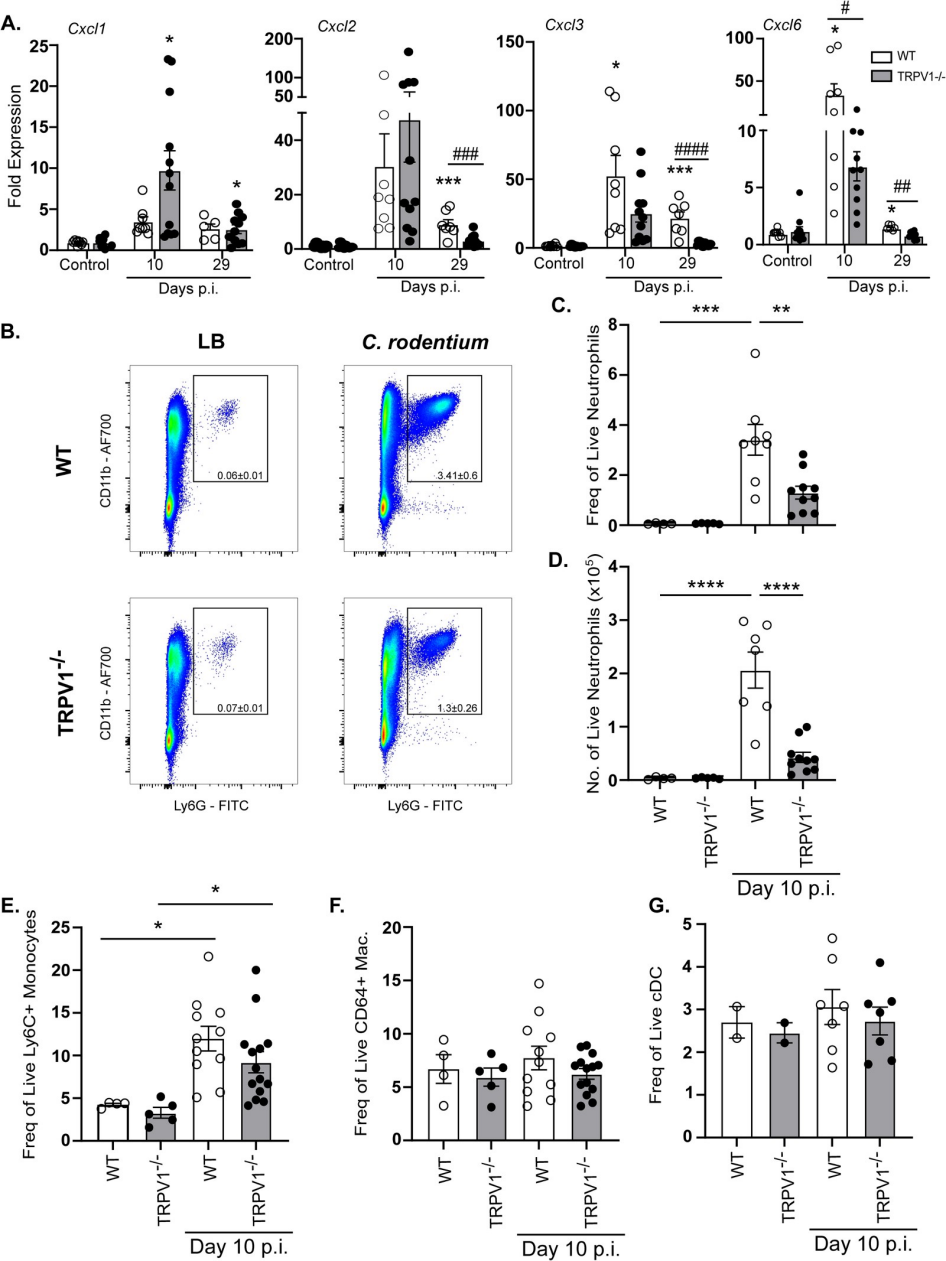
Neutrophil recruitment is impaired in TRPV1^-/-^ mice during *C*. *rodentium* infection. **(A)** Common neutrophil chemokines *Cxcl1*, *Cxcl2*, *Cxcl3*, and *Cxcl6* were assessed by qPCR performed on colonic tissue from control or *C*. *rodentium* infected wild-type (WT) or TRPV1^-/-^ mice. **(B-D)** Neutrophil recruitment was determined by flow cytometry for live CD45^+^ CD11b^+^ Ly6G^+^ cells in the whole colon after epithelial cells were removed from WT and TRPV1^-/-^ mice given vehicle control (LB) and 10 days p.i. with *C*. *rodentium* **(B)** Representative flow plots and cumulative data of **(C)** frequency of live neutrophils and **(D)** total cell number of live neutrophils in the colon. **(E-G)** Cumulative data of frequency of live **(E)** Ly6C^+^ monocytes, **(F)** CD64^+^ macrophages, and **(G)** CD11c^hi^ conventional dendritic cells (cDC) in the lamina propria of the colon of WT and TRPV1^-/-^ mice at baseline and 10 days p.i. Data are presented as mean ± standard error of the mean: *, P < 0.05, **, P < 0.01 and ***, P < 0.001 compared with uninfected mice of the same genotype; #, P < 0.05 compared with WT *C*. *rodentium*-infected mice at the same time point **(A)** *, P < 0.05, **, P < 0.01, and ***, P < 0.0001 **(C-G)**; one-way ANOVA with post-hoc analysis using Tukey’s multiple comparisons test. 4–11 animals per group. LB, Luria-Bertani; p.i., post-infection.

To determine the effect of TRPV1 on neutrophil recruitment to the colon during *C*. *rodentium* infection, we first assessed colonic expression of chemokines in TRPV1^-/-^ and WT mice. Expression of *Cxcl1* was significantly increased at 10 days p.i. irrespective of genotype. Similarly, *Cxcl2 and Cxcl3* expression were increased during infection; however, there was a significant defect in TRPV1^-/-^ mice to express these chemokines 29 days p.i. compared to WT mice. TRPV1^-/-^ mice also failed to produce *Cxcl6*, a critical neutrophil chemokine and antimicrobial peptide [34], compared to WT mice at 10- and 29- days p.i. **(Fig 4A)**. Given these significant differences in chemokines that recruit neutrophils, and reduced colonic *Cxcr2*, we assessed neutrophil recruitment to the colonic lamina propria during *C*. *rodentium* infection in TRPV1^-/-^ and WT mice.

Flow cytometry on colonic lamina propria demonstrated a significant failure of *C*. *rodentium* infection to elicit neutrophil recruitment in TRPV1^-/-^ mice compared to WT at 10 days p.i. **(Fig 4B–4D)**; however, other innate immune cell populations such as monocytes, macrophages, or conventional dendritic cells were not impacted **(Fig 4E–4G)**. Despite differences in *Cxcl6*, neutrophil chemokines are often redundant and conserved, so loss of one may not impact neutrophil chemotaxis entirely on its own [35,36]. To determine if the significant reductions in colonic neutrophils were due to reduced differentiation, pre-neutrophil, immature neutrophil, and mature neutrophil subsets within the bone marrow at baseline and 10 days p.i. were assessed and found no significant difference between WT and TRPV1^-/-^ mice **(S5B Fig)**. Together, these data demonstrate that the TRPV1 induced reduction of colonic neutrophil recruitment during *C*. *rodentium* infection is not due to cell maturation.

### TRPV1 regulates colonic blood endothelial cell expression of selective adhesion molecules during *C*. *rodentium* infection

**Fig 5:**
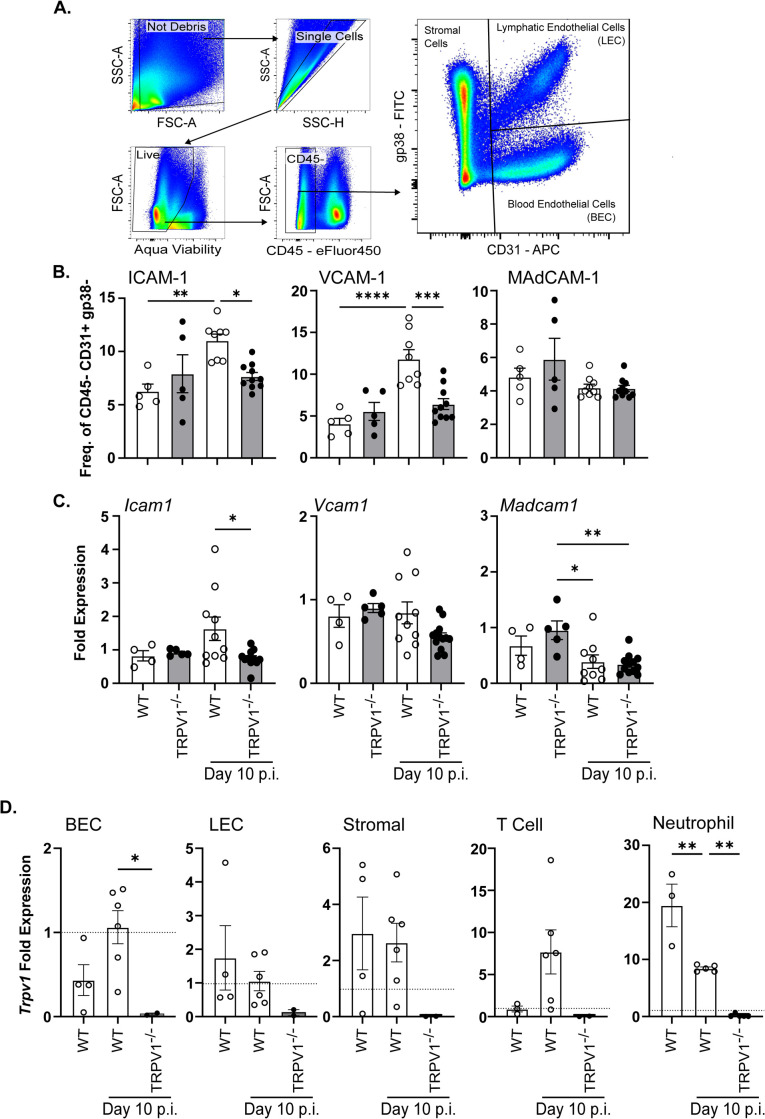
TRPV1 dependent neutrophil recruitment is driven by upregulation of ICAM-1 and VCAM-1 on blood endothelial cells. **(A & B)** Colonic blood endothelial cells from wild-type (WT) and TRPV1^-/-^ mice were assessed for their expression of ICAM-1, VCAM-1, and MAdCAM-1 by flow cytometry at baseline and 10 days p.i. **(C)** *Icam1*, *Vcam1*, and *Madcam1* mRNA were assessed by qPCR of the colonic tissue at baseline and 10 days p.i. **(D)** Sorted blood endothelial cells (BEC), lymphatic endothelial cells (LEC), stromal cells, CD3^+^ T cells, and Ly6G^+^ neutrophils from colons of WT LB and WT and TRPV1-/- mice 10 days p.i. with *C*. *rodentium* were assessed for their expression of *Trpv1* via a cDNA primer set specific for the deletion in TRPV1^-/-^ mice. Expression is relative to β-actin in each sample and normalized to vagal ganglion from a WT mouse. Data are presented as mean ± standard error of the mean: *, P < 0.05, **, P < 0.01 and ***, P < 0.001; one-way ANOVA with post-hoc analysis using Tukey’s multiple comparisons test. 4–10 animals per group. LB, Luria-Bertani; p.i., post-infection.

**Fig 6:**
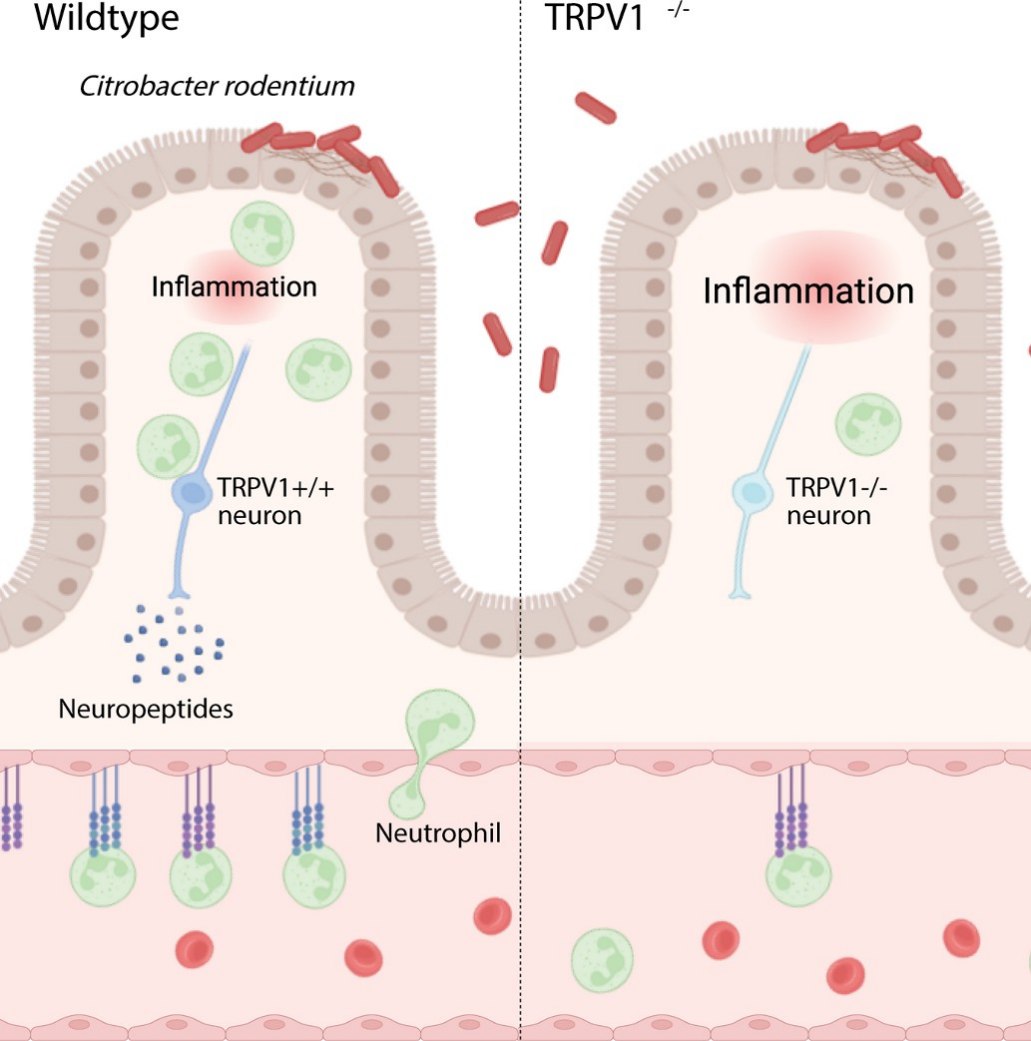
Neuronal TRPV1 signaling coordinates host protective immune responses during *C*. *rodentium* infection. TRPV1^+^ neurons promote the recruitment of neutrophils by upregulating cell adhesion molecules, ICAM-1 and VCAM-1, on blood endothelial cells and by promoting the release of the chemokine, CXCL6. These factors collectively promote extravasation of neutrophils from the blood vessel into the site of infection for clearance of *C*. *rodentium*. Mice lacking TRPV1 exhibit dramatic decreases in recruitment of neutrophils necessary for *C*. *rodentium* clearance from the colon. Created with BioRender.com.

Considering the reduced numbers of colonic neutrophils that were not due to development of neutrophils in bone marrow, we assessed if TRPV1^-/-^ mice had deficiencies in the processes that regulated neutrophil recruitment. Using flow cytometry, *C*. *rodentium* infection (10 days p.i.) significantly increased ICAM-1 and VCAM-1 on the surface of colonic lamina propria blood endothelial cells (BEC; single, live, CD45^-^, CD31^+^, gp38^-^) in WT but not in TRPV1^-/-^ mice. Infection induced changes in adhesion molecule expression were not global, as MAdCAM-1^+^ BECs were not different in WT or TRPV1^-/-^ mice. **(Fig 5A and 5B)**. Quantification of mRNA transcripts by qPCR for these cell adhesion molecules revealed that *Icam1* but not *Vcam1* or *Madcam1* was significantly increased at 10 days p.i. in WT mice while TRPV1^-/-^ mice failed to elicit a similar response to *C*. *rodentium* **(Fig 5C)**. Given the impact TRPV1 has on BECs and neutrophil recruitment, we assessed the expression of *Trpv1* mRNA transcripts on these cell populations from the colon. Sorted colonic BECs, lymphatic endothelial cells (LEC), and stromal cells express *Trpv1* mRNA at baseline with infection induced upregulation of *Trpv1* in BECs **(Fig 5D)**. Additionally, sorted colonic T cells do not appear to express *Trpv1* at baseline, however, *C*. *rodentium* infection increased *Trpv1* expression. Colonic neutrophils also exhibited high levels of *Trpv1* transcripts that were downregulated upon infection **(Fig 5D)**. Together these data suggest that TRPV1 regulates the recruitment of immune cells through regulation of adhesion molecules expressed by blood endothelial cells in the colon **(Fig 6)**.

## Discussion

Host immune responses to enteric bacterial pathogens are highly complex, requiring coordination between immune, epithelial, and stromal cells. Integral to these responses is the neuronal innervation of the intestinal tract, and the ability of these neurons to communicate with these various cell types through the release of specific neurotransmitters. Previously, we demonstrated a critical role for TRPV1^+^ sensory nociceptive neurons in the control of *C*. *rodentium* infection by ablation of these neurons [11]; however, it was unclear if these host-protective effects were dependent on TRPV1 specifically, or by other receptors or secreted factors produced by these TRPV1^+^ neurons. Indeed, deficiency in the related transient receptor potential cation channel, subfamily A, member 1 (TRPA1), which could also be expressed by these intestinal nociceptive neurons, significantly increased *C*. *rodentium* burden and tissue pathology [37].

Using WT and TRPV1^-/-^ mice, we now demonstrate that this polymodal nociceptor is a critical component of the host response to enteric bacterial infection with *C*. *rodentium*. Deficiency in TRPV1 not only increased bacterial burden in the feces and colon, but also exacerbated colonic histopathology. Intestinal epithelial cells have been reported to express TRPV1 [27,28], and it has further been suggested that TRPV1 negatively regulates IEC proliferation [22]. Although non-infected TRPV1^-/-^ mice exhibited increased crypt length compared to WT, the number of proliferating cells, indicated by Ki67 staining, was not different in uninfected mice. Moreover, epithelial cell organoid cultures where proliferation was measured by incorporation of the thymidine analog EdU was not different in WT or TRPV1^-/-^ derived cultures, and this rate of proliferation was not altered by treatment with the TRPV1 agonist capsaicin. Together these findings suggest that the increased crypt length and IEC proliferation observed in TRPV1^-/-^ compared to WT mice, was due to increased bacterial burden and the ensuing immune response, as opposed to altered IEC intrinsic TRPV1 signaling **(Fig 6)**.

As a major component of the host response to *C*. *rodentium* infection depends on CD4^+^ T cells, we assessed the effect of TRPV1 deficiency in the recruitment of these cells. No significant difference in colonic T cells was observed in uninfected or *C*. *rodentium* infected TRPV1^-/-^ compared to WT mice. Quantification of the cytokines produced by colonic lamina propria T cells revealed only subtle but significantly reduced IL-17A production in infected TRPV1^-/-^ mice compared to WT. Previous reports found that during DSS induced colitis, mice with a mutation to constitutively activate TRPV1 showed a significant increase in CD4^+^ T cells producing IL-17A but not IFNγ [38]. This was attributed to an increased pro-inflammatory state of DC, but here we show that in a genetic knockout of TRPV1, IL-17A was significantly decreased in CD4^+^ T cells. These observations are in keeping with reduced expression of IL-17A in TRPV1^-/-^ T cells *in vitro*, although in contrast to that study we found no difference in IFNγ expression in CD4^+^ T cells *in vivo* [21]. We also found no difference in *in vitro* T cell proliferation induced by TCR activation with or without co-stimulation, and no effect of the TRPV1 agonist capsaicin on proliferation in TRPV1^-/-^ compared to WT cells. These differences are most likely attributed to our analysis after *in vivo* infection compared to an *in vitro* assay with purified T cells. Although it is unclear what TRPV1 agonists would be present in an *in vitro* culture, these differences could also be attributed to unique ligands present during infection-induced inflammation *in vivo* compared to *in vitro*. In addition, since most *in vitro* assays utilize splenic T cells, it is unclear if expression of TRPV1 in CD4^+^ T cells is retained after recruitment to the lamina propria as an effector cell. Our qPCR data suggests that recruited colonic T cells during *C*. *rodentium* infection upregulate *Trpv1* mRNA transcripts compared to LB treated mice; however, it is unclear if TRPV1 protein on T cells is altered during infection. Further research identifying the factors produced under homeostasis and during inflammation that regulate TRPV1 in T cells are required. Although adaptive immune responses are critical for the clearance of *C*. *rodentium* infection and sterilizing immunity [32], there are critical and non-redundant roles for innate immune cells. These innate immune responses include bactericidal activity, phagocytosis, and release of chemokines and cytokines. During enteric bacterial infection, chemokine and cytokine production serve to increase the expression of host protective factors such as antimicrobial peptides and to increase blood endothelial cell adhesion molecules. Enteric infection resulted in significantly increased gene expression of cytokines including *Il6*, *Tnfα* and *Il1β*; however, at 10 days p.i., TRPV1^-/-^ mice failed to upregulate *Il1β* compared to WT while *Il6* and *Tnfα* were not different between TRPV1^-/-^ and WT mice. Additionally, at 29 days p.i., only *Il6* was significantly increased in TRPV1^-/-^ mice compared to WT; however, very little IL-6 was detected in the serum at day 29 p.i. While the expression of the antimicrobial protein *RegIIIγ* was significantly increased in infected TRPV1^-/-^ compared to WT mice, this is likely a response to the significantly increased bacterial burden in these animals.

Neutrophils have been previously demonstrated to be indispensable antibacterial effectors during enteric bacterial infection. Depletion, or genetic deficiency in chemokine receptors that dictate homing and migration of these cells into infected tissues, resulted in significantly increased bacterial burden and pathology [16]. Our flow cytometric analysis revealed significantly reduced numbers of colonic neutrophils in *C*. *rodentium* infected TRPV1^-/-^ mice which is consistent with a role for TRPV1 in promoting host protective recruitment of these cells. Despite reduced recruitment of these cells, only *Cxcl6*, a neutrophil chemokine and antimicrobial peptide produced by activated resident macrophages and epithelial cells [34], and IL-17A were reduced in infected TRPV1^-/-^ mice. However, chemokines are well established to have compensatory roles, where deficiency in one chemokine is unlikely to result in a drastic phenotype [35,36]. Our analysis on the development of neutrophils from the granulocyte monocyte progenitor in the bone marrow of control and infected WT and TRPV1^-/-^ mice demonstrated no impact in development and maturation of these cells. These data indicate that any deficiency in the colonic tissue is therefore not simply due to a reduced ability to produce neutrophils in the absence of TRPV1. In support of this finding, prior studies with TRPV1^+^ neuronal ablation also showed no difference in the production of many immune cell types including neutrophils [39]. Our data further shows neutrophils express high levels of *Trpv1* mRNA at baseline which decreases 10 days p.i. Others have also shown that neutrophils express TRPV1 at the mRNA level [40], suggesting that TRPV1 dependent recruitment could be a neutrophil intrinsic effect; however, the expression of TRPV1 protein and any functional role of this protein remains unclear. Prior publications demonstrate capsaicin induced Ca^2+^ flux in human neutrophils at high concentrations and was TRPV1-independent [41], raising questions as to the functional expression of TRPV1 [31,42]. Additional research is needed to understand what role, if any, TRPV1 has on neutrophil function.

Recruitment of immune cells and extravasation into the infected tissue is dependent on the interaction of specific adhesion molecules on the immune and blood endothelial cells [43–46]. Adhesion molecule expression and localization on the luminal surface of endothelial cells is well appreciated to increase during inflammation due to cytokines such as TNFα and IL-1β [47–49], prostaglandins [50], and bacterial products such as LPS [47,51]. Cytokines and products released during inflammation are not the only factors that can activate endothelial cells to promote recruitment of immune cells from the blood. Neurogenic inflammation is due to neurotransmitters released from TRPV1^+^ sensory neurons such as SP and CGRP acting on blood endothelial cells which increase blood vessel permeability and ICAM-1 on the endothelial cell surface [7,10,52]. Our data demonstrate that colonic endothelial cells from *C*. *rodentium* infected TRPV1^-/-^ mice have significantly reduced ICAM-1 and VCAM-1 expression compared to WT mice **(Fig 6)**. These findings agree with prior publications showing that the blockade of ICAM-1 attenuated colonic neutrophil recruitment during *C*. *rodentium* infection [43]. Additionally, it has been shown that the neutrophil-recruiting chemokine CXCL6 is upregulated by TRPA1 signaling [53], supporting regulation of this chemokine within the colon in a TRP channel dependent manner.

TRPV1 deletion impacts critical processes for maintaining the bacterial burden during *C*. *rodentium*, but it remains unclear if it is a cell intrinsic effect or neuronally driven. Here, we show that blood endothelial, lymphatic endothelial and stromal cells express *Trpv1* mRNA, suggesting there may be a cell intrinsic effect of TRPV1 signaling in these populations to control neutrophil recruitment. Despite these data, TRPV1 immunostaining in the colon showed very little non-neuronal specific protein expression, suggesting that either the largest effect of TRPV1 deletion is due to loss of function on nociceptors or that different isoforms of this protein may exist that are not detected by many commercially available antibodies. Direct inflammatory effects of TRPV1^+^ neuronal activation have also been observed previously by selective activation of cutaneous sensory nociceptors using optogenetics to induce localized inflammation. Of particular interest, stimulation of these nerves induced IL-23 dependent recruitment of neutrophils and Th17 CD4^+^ T cells [54]. Neuronal TRPV1 dependent recruitment of neutrophils has also been shown during TLR agonist induced skin inflammation [39]. In this study, the ablation of TRPV1 neurons had attenuated inflammation which was attributed to extravascular mechanisms; however, it is critical to note that adhesion molecule expression of leukocyte rolling was not assessed during inflammation. In this context, our data suggests that TRPV1^+^ sensory neurons have a conserved ability to regulate inflammation in the intestinal tract. This ability of nociceptive neurons to control neutrophil recruitment in the intestinal tract is likely to have unique outcomes depending on the health of the host and the specific challenges that elicit this effect. In mouse models of colitis, neutrophils can exert host beneficial [55,56] or detrimental effects [57,58] depending on the model of disease. With these differential effects, it is tempting to speculate that this may be the basis for seemingly contradictory roles of nociceptive neuropeptides. As such, there appears to be considerable biological complexity, where sensory innervation, the signals released by these neurons, and the cells targeted may be capable of eliciting unique outcomes and suggests that, with further understanding, these processes could be used to enhance or reduce inflammation in a contextually meaningful manner. For example, treatment-resistant patients with IBD exhibited increased levels of neutrophils that correlated with increased expression of IL-1β and CXCL6 [59]. Since TRPV1 has been implicated in gastrointestinal disorders including IBD [60,61], our data suggests a role for modulation of TRPV1 activity in the intestine beyond nociception, to regulate inflammation in a host beneficial manner that should be explored further.

## Supporting information

S1 Fig TRPV1 expressed in the colon does not affect gut permeability or epithelial cell proliferation. **(A)** Paraffin embedded colonic tissue of wild-type (WT) and TRPV1^-/-^ mice was stained with anti-TRPV1 (red), anti-βIII tubulin (green), and DAPI (blue). **(B)** WT (open circles) and TRPV1^-/-^ (black circles) mice colons were assessed for gut permeability through Ussing chambers. Not significant. Student t test with 7–9 animals per group. **(C)** Proliferation of colon-derived organoids was assessed *in vitro* with EdU incorporation and diameter measurements. WT and TRPV1^-/-^ derived organoids were compared. Not significant. Student t test comparing WT to TRPV1^-/-^ with 7–11 organoids from 3 mice per group.
(TIF)

S2 Fig IFNγ, IL-17A, and IL-22 show no difference in mRNA transcripts at baseline and 10- and 29- days post-infection by *C*. *rodentium*. **(A-C)** Colonic tissue from wild-type (WT) and TRPV1^-/-^ mice was assessed by qPCR for expression of common T cell produced cytokines relevant to *C*. *rodentium* clearance such as **(A)** *Ifnγ*, **(B)** *Il17a*, and **(C)** *Il22*. Data are presented as mean ± standard error of the mean: *, P < 0.05, **, P < 0.01 and ***, P < 0.001; one-way ANOVA with post-hoc analysis using Tukey’s multiple comparisons test. 6–12 animals per group. **(D & E)** WT or TRPV1^-/-^ negatively selected CD3^+^ CD4^+^ T cells were stained with CellProliferation dye eFluor450 and cultured *in vitro* at described concentrations of anti-CD3ε and anti-CD28 antibodies for 72 hours and then analyzed by flow cytometry for proliferation index. **(D)** Comparison of WT and TRPV1^-/-^ T cell proliferation at different concentrations of anti-CD3ε and anti-CD28 antibodies. **(E)** WT T cells were cultured with 1 μg/mL of anti-CD3ε and 1 μg/mL of anti-CD28 and a dose response of the TRPV1 agonist capsaicin. After 72 hours, cells were analyzed by flow cytometry for proliferation index. Data are presented as mean ± standard error of the mean: not significant.
(TIF)

S3 Fig Lamina propria T cell gating strategy. Whole colon dissociated into a single cell suspension and stained for antibodies to identify live CD45^+^ CD3^+^ CD4^+^ T cells.
(TIF)

S4 Fig Serum Levels of IL-6. Serum from wild-type (WT) and TRPV1^-/-^ mice at baseline (control treated with LB) and 10- and 29- days p.i. was analyzed for IL-6 by ELISA. Data are presented as mean ± standard error of the mean: one-way ANOVA with Tukey post-test, with 8–14 animals per group, not significant. LB, Luria-Bertani; p.i., post-infection.
(TIF)

S5 Fig TRPV1 deletion did not alter neutrophil precursors in the bone marrow compartment. Wild-type (WT) and TRPV1^-/-^ mice had right femur bone marrow extracted at baseline and 10 days p.i. of *C*. *rodentium* and stained for pre-neutrophils (SiglecF^-^, CD115^-^, Gr-1^+^, CD11b^+^, CXCR4^+^), immature neutrophils (SiglecF^-^, CD115^-^, Gr-1^+^, CD11b^+^, CXCR4^-^, CXCR2^-^), and mature neutrophils (SiglecF^-^, CD115^-^, Gr-1^+^, CD11b^+^, CXCR4^-^, CXCR2^+^, Ly6G^+^). **(A)** Bone marrow gating strategy and **(B)** frequency of live of each subset of neutrophil lineage shown. Data are presented as mean ± standard error of the mean: one-way ANOVA with Tukey post-test, with 7–12 animals per group, not significant. **(C)** Gating strategy for whole colon dissociated into a single cell suspension and stained for antibodies to identify live (CD45^+^, Ly6G^+^, CD11b^+^) neutrophils, (CD45^+^, Ly6G^-^, Ly6C^+^) monocytes, (CD45^+^, Ly6G^-^, Ly6C^-^, CD64^+^) macrophages, (CD45^+^, Ly6G^-^, Ly6C^-^, CD64^-^, CD11c^hi^) conventional dendritic cells (DC).
(TIF)

S1 Table Primers used in this study. (DOCX)

S2 Table Antibodies used for confocal microscopy. (DOCX)

S3 Table Antibodies used for flow cytometry. (DOCX)
