# Supplementary figures and images for: TRPV1 controls innate immunity during Citrobacter rodentium enteric infection
Source: PLoS Pathog. 2023 Dec 18;19(12):e1011576. doi: 10.1371/journal.ppat.1011576 (PMC10758261; doi:10.1371/journal.ppat.1011576)

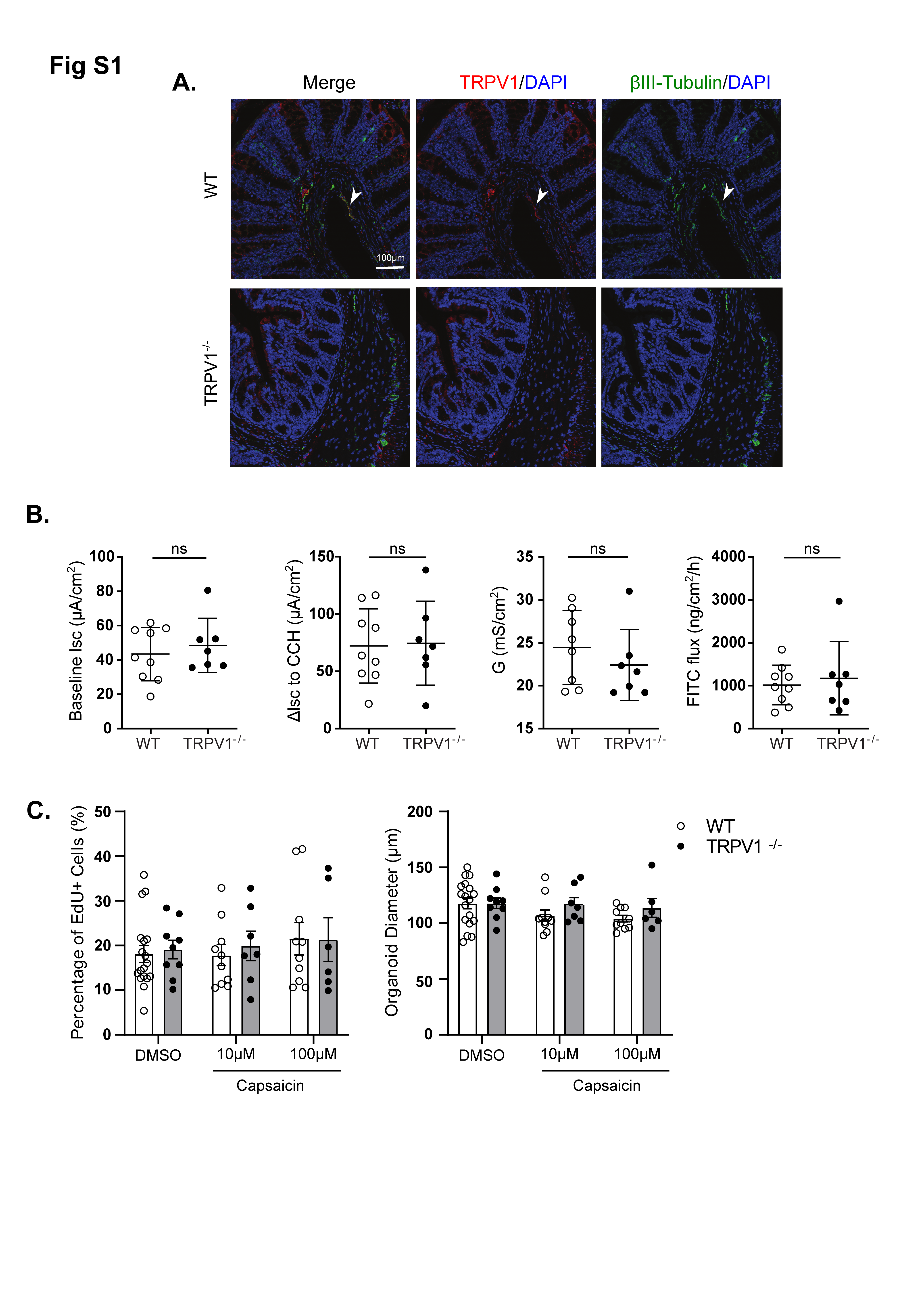

Supplement: S1 Fig — (A) Paraffin embedded colonic tissue of wild-type (WT) and TRPV1-/- mice was stained with anti-TRPV1 (red), anti-βIII tubulin (green), and DAPI (blue). (B) WT (open circles) and TRPV1-/- (black circles) mice colons were assessed for gut permeability through Ussing chambers. Not significant. Student t test with 7–9 animals per group. (C) Proliferation of colon-derived organoids was assessed in vitro with EdU incorporation and diameter measurements. WT and TRPV1-/- derived organoids were compared. Not significant. Student t test comparing WT to TRPV1-/- with 7–11 organoids from 3 mice per group. (TIF) [file ppat.1011576.s001.tif]

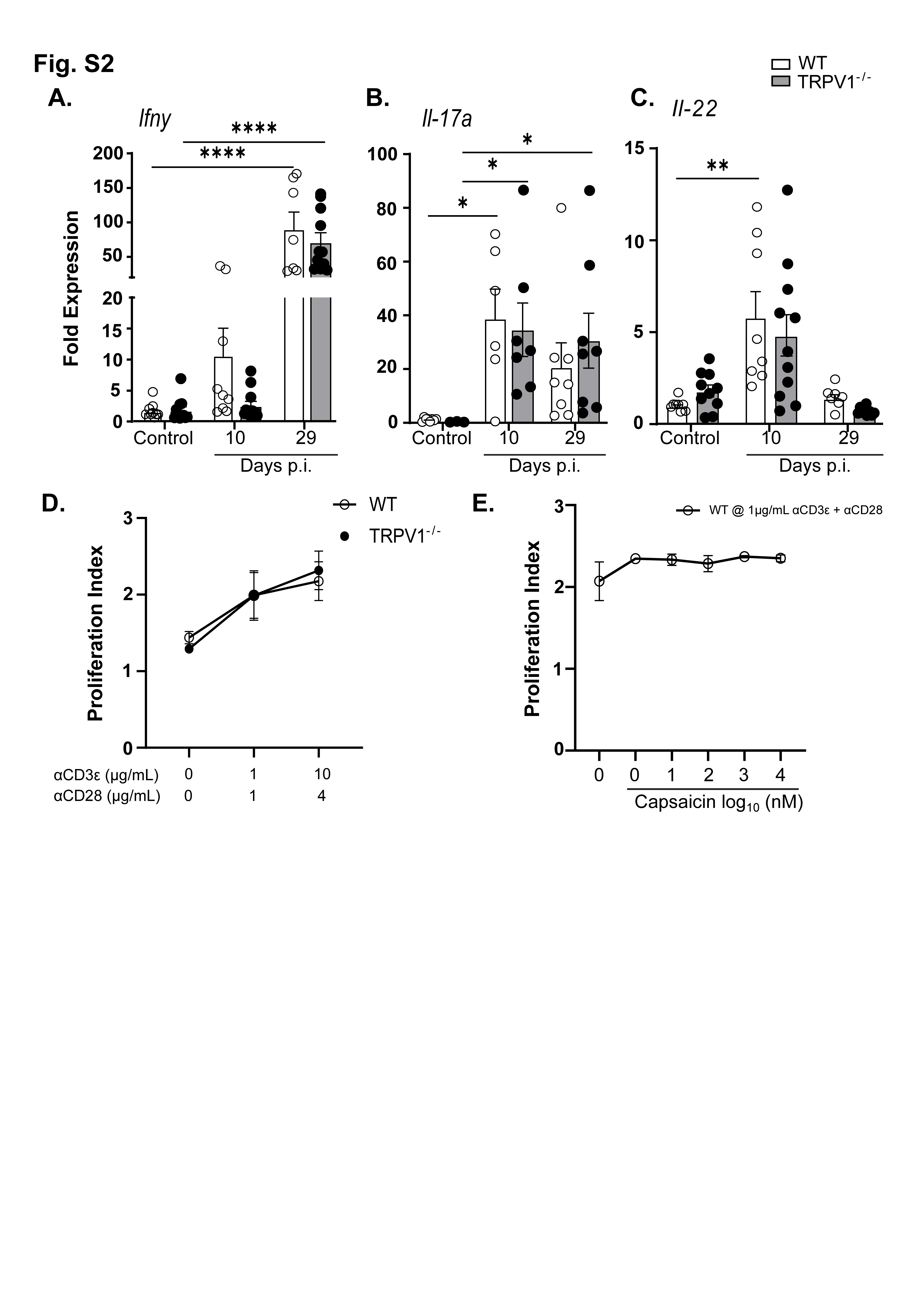

Supplement: S2 Fig — (A-C) Colonic tissue from wild-type (WT) and TRPV1-/- mice was assessed by qPCR for expression of common T cell produced cytokines relevant to C. rodentium clearance such as (A) Ifnγ, (B) Il17a, and (C) Il22. Data are presented as mean ± standard error of the mean: *, P < 0.05, **, P < 0.01 and ***, P < 0.001; one-way ANOVA with post-hoc analysis using Tukey’s multiple comparisons test. 6–12 animals per group. (D & E) WT or TRPV1-/- negatively selected CD3+ CD4+ T cells were stained with CellProliferation dye eFluor450 and cultured in vitro at described concentrations of anti-CD3ε and anti-CD28 antibodies for 72 hours and then analyzed by flow cytometry for proliferation index. (D) Comparison of WT and TRPV1-/- T cell proliferation at different concentrations of anti-CD3ε and anti-CD28 antibodies. (E) WT T cells were cultured with 1 μg/mL of anti-CD3ε and 1 μg/mL of anti-CD28 and a dose response of the TRPV1 agonist capsaicin. After 72 hours, cells were analyzed by flow cytometry for proliferation index. Data are presented as mean ± standard error of the mean: not significant. (TIF) [file ppat.1011576.s002.tif]

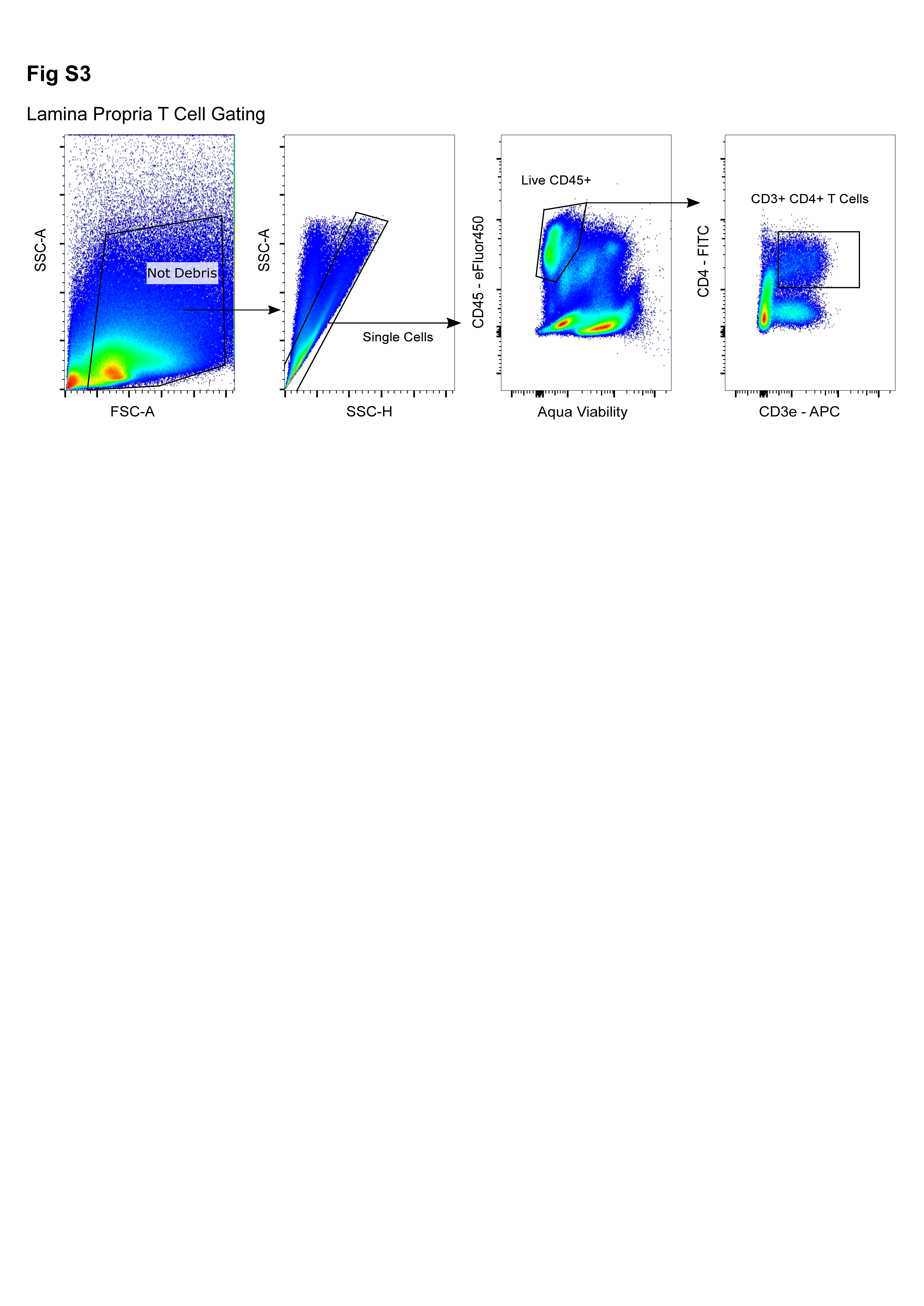

Supplement: S3 Fig — Whole colon dissociated into a single cell suspension and stained for antibodies to identify live CD45+ CD3+ CD4+ T cells. (TIF) [file ppat.1011576.s003.tif]

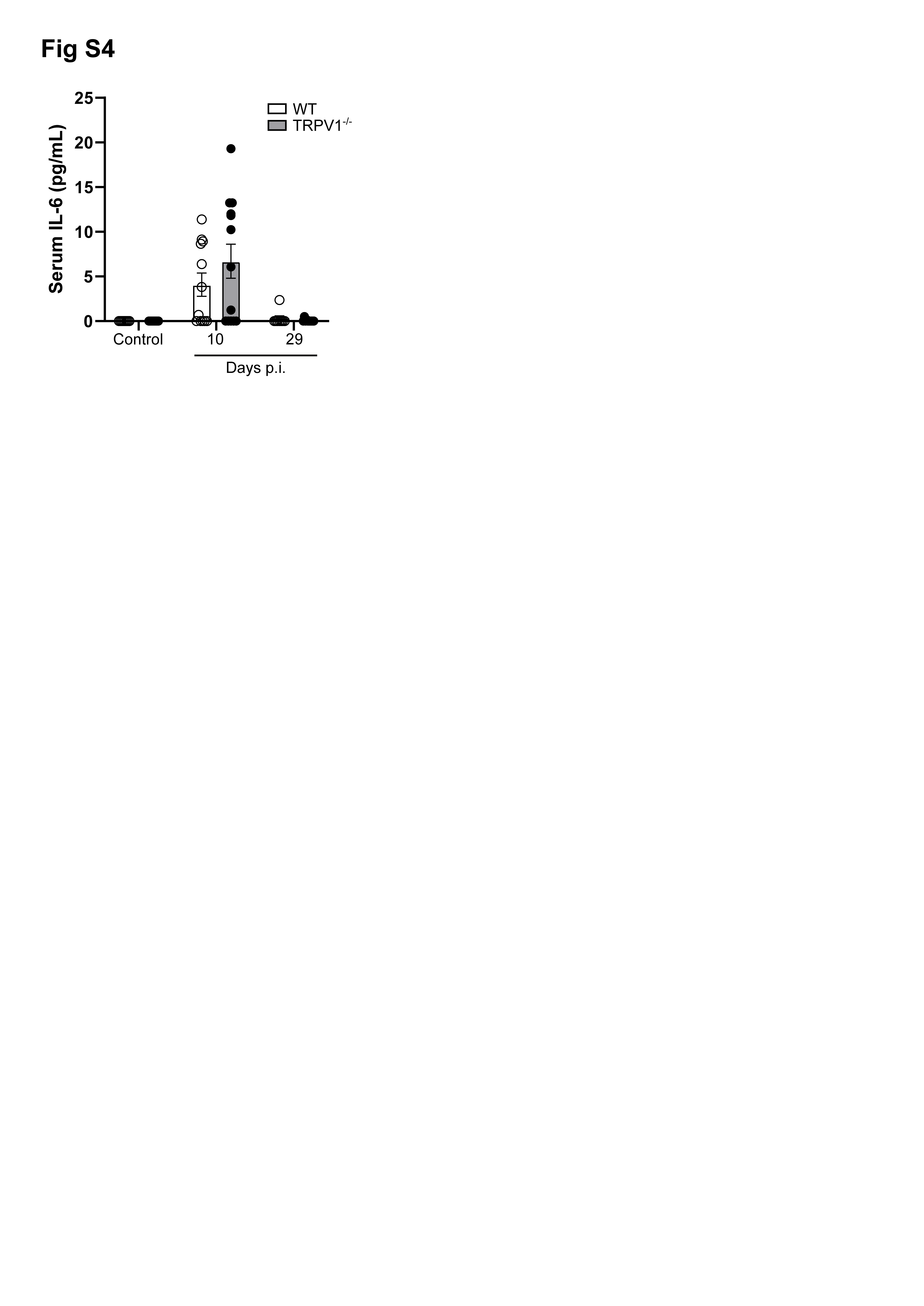

Supplement: S4 Fig — Serum from wild-type (WT) and TRPV1-/- mice at baseline (control treated with LB) and 10- and 29- days p.i. was analyzed for IL-6 by ELISA. Data are presented as mean ± standard error of the mean: one-way ANOVA with Tukey post-test, with 8–14 animals per group, not significant. LB, Luria-Bertani; p.i., post-infection. (TIF) [file ppat.1011576.s004.tif]

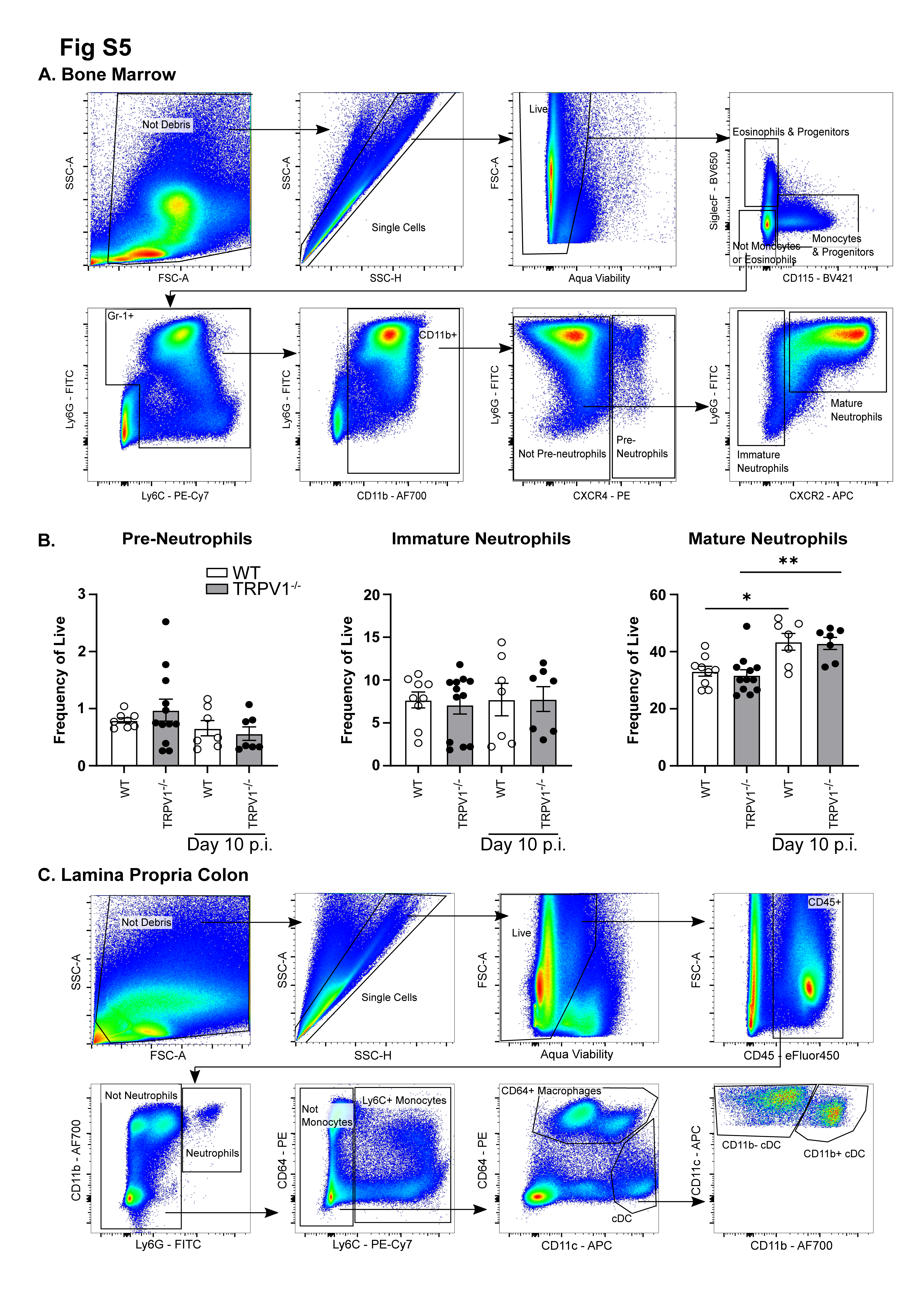

Supplement: S5 Fig — Wild-type (WT) and TRPV1-/- mice had right femur bone marrow extracted at baseline and 10 days p.i. of C. rodentium and stained for pre-neutrophils (SiglecF-, CD115-, Gr-1+, CD11b+, CXCR4+), immature neutrophils (SiglecF-, CD115-, Gr-1+, CD11b+, CXCR4-, CXCR2-), and mature neutrophils (SiglecF-, CD115-, Gr-1+, CD11b+, CXCR4-, CXCR2+, Ly6G+). (A) Bone marrow gating strategy and (B) frequency of live of each subset of neutrophil lineage shown. Data are presented as mean ± standard error of the mean: one-way ANOVA with Tukey post-test, with 7–12 animals per group, not significant. (C) Gating strategy for whole colon dissociated into a single cell suspension and stained for antibodies to identify live (CD45+, Ly6G+, CD11b+) neutrophils, (CD45+, Ly6G-, Ly6C+) monocytes, (CD45+, Ly6G-, Ly6C-, CD64+) macrophages, (CD45+, Ly6G-, Ly6C-, CD64-, CD11chi) conventional dendritic cells (DC). (TIF) [file ppat.1011576.s005.tif]
