# Supplementary material for: TRPV1 controls innate immunity during Citrobacter rodentium enteric infection
Source: PLoS Pathog. 2023 Dec 18;19(12):e1011576. doi: 10.1371/journal.ppat.1011576 (PMC10758261; doi:10.1371/journal.ppat.1011576)
Supplement: S1 Table — (DOCX) [file ppat.1011576.s006.docx]

Table S1. List of primers used for qPCR

| **Target** | **Forward 5’-3’** | **Reverse 5’-3’** |
| --- | --- | --- |
| *Il1β* | CTGTGACTCATGGGATGATGATG | CGGAGCCTGTAGTGCAGTTG |
| *Il6* | TAGTCCTTCCTACCCCAATTTCC | TTGGTCCTTAGCCACTCCTTC |
| *Il17a* | TTTAACTCCCTTGGCGCAAAA | CTTTCCCTCCGCATTGACAC |
| *Il22* | ATGAGTTTTTCCCTTATGGGGAC | CTGGAAGTTGGACACCTCAA |
| *Ifny* | GCCACGGCACAGTCATTGA | TGCTGATGGCCTGATTGTCTT |
| *Tnfa* | CCCTCACACTCAGATCATCTTCT | GCTACGACGTGGGCTACAG |
| *Nos2* | GTTCTCAGCCCAACAATACAAGA | GTGGACGGGTCGATGTCAC |
| *RegIIIy* | CCTCAGGACATCTTGTGTC | TCCACCTCTGTTGGGTTCA |
| *Icam1* | GTGATGCTCAGGTATCCATCCA | CACAGTTCTCAAAGCACAGCG |
| *Vcam1* | AGTTGGGGATTCGGTTGTTCT | CCCCTCATTCCTTACCACCC |
| *Madcam1* | CCTGGCCCTAGTACCCTACC | CCGTACAGAGAGGATACTGCTG |
| *Cxcr2* | ATGCCCTCTATTCTGCCAGAT | GTGCTCCGGTTGTATAAGATGAC |
| *Cxcl1* | TCCAGAGCTTGAAGGTGTTGCC | AACCAAGGGAGCTTCAGGGTCA |
| *Cxcl2* | CTCTCAAGGGCGGTCAAAAAGTT | TCAGACAGCGAGGCACATCAGGTA |
| *Cxcl3* | CATCCAGAGCTTGACGGTGA | ACACATCCAGACACCGTTGG |
| *Cxcl6* | TGGATCCAGAAGCTCCTGTGA | TGCATTCCGCTTAGCTTTCTTT |
| *Trpv1* | TAGTGACACTGATCGAGGATGG | GCCGATGGTGAACTTGAACAG |
| *β-actin* | GGCTGTATTCCCCTCCATCG | CCAGTTGGTAACAATGCCATGT |
