# Supplementary material for: TRPV1 controls innate immunity during Citrobacter rodentium enteric infection
Source: PLoS Pathog. 2023 Dec 18;19(12):e1011576. doi: 10.1371/journal.ppat.1011576 (PMC10758261; doi:10.1371/journal.ppat.1011576)
Supplement: S2 Table — (DOCX) [file ppat.1011576.s007.docx]

Table S2. Antibodies used for Confocal Imaging

| **Target** | **Host** | **Source** | **Catalog No** | **Dilution** |  |
| --- | --- | --- | --- | --- | --- |
| CD3 | Rat | Bio-Rad | CD3-12 | 1:200 |  |
| βIII Tubulin | Mouse | ThermoFisher | MA1-118 | 1:5000 |  |
| CDH1 | Mouse | ECM Bioscience | CP1921 | 1:300 |  |
| Ki67 | Rabbit | LSBio | LS-C141898 | 1:600 |  |
| TRPV1 | Rabbit | Alomone Labs | ACC-030 | 1:200 |  |
| **Target** | **Host** | **Conjugate** | **Source** | **Catalog No** | **Dilution** |
| Streptavidin |  | Alexa Fluor 488 | ThermoFisher | S32354 | 1:200 |
| Anti-rabbit | Goat | Alexa Fluor 488 | ThermoFisher | A32732 | 1:200 |
| Anti-rat | Donkey | Alexa Fluor 647 | Abcam | Ab150155 | 1:200 |
