# Supplementary material for: TRPV1 controls innate immunity during Citrobacter rodentium enteric infection
Source: PLoS Pathog. 2023 Dec 18;19(12):e1011576. doi: 10.1371/journal.ppat.1011576 (PMC10758261; doi:10.1371/journal.ppat.1011576)
Supplement: S3 Table — (DOCX) [file ppat.1011576.s008.docx]

Table S3: Flow cytometry antibodies

| **Antigen Target** | **Manufacture & Clone** | **Catalog number** |
| --- | --- | --- |
| CD3 | Tonbo Biosciences 145-2C11 | 20-0031 |
| CD4 | BD Biosciences RM4-5 | 553047 |
| CD45 | Invitrogen 30-F11 | 48-0451-82 |
| Ly6G | Tonbo Biosciences 1A8 | 35-1276 |
| Ly6G | Invitrogen 1A8-Ly6g | 12-9668-82 |
| CD11b | Invitrogen M1/70 | 56-0112-82 |
| IFNγ | Invitrogen XMG1.2 | 25-7311-82 |
| IL-17A | BD Biosciences TC11-18H10 | 561020 |
| IL-22 | Invitrogen 1HBPWSR | 46-7221-82 |
| CD31 | Biolegend MEC13.3 | 102533 |
| gp38 | Biolegend 8.1.1 | 127405 |
| ICAM-1 | Biolegend YN1/1.7.4 | 116114 |
| VCAM-1 | Biolegend 429(MVCAM.A) | 105719 |
| MAdCAM-1 | Biolegend MECA-367 | 120710 |
| Fixable Live/Dead Aqua | ThermoFisher | L34957 |
| CD115 | BD Bioscience T38-320 | 743638 |
| SiglecF | BD Biosciences E50-2440 | 740557 |
| Ly6C | Invitrogen HK1.4 | 25-5932-82 |
| CXCR4 | Miltenyi Biotec REA107 | 130-118-682 |
| CXCR2 | Miltenyi Biotec REA942 | 130-115-635 |
| CD64 | Miltenyi Biotech REA286 | 130-118-684 |
| CD11c | Invitrogen N418 | 17-0114-81 |
